# Rickettsioses as Major Etiologies of Unrecognized Acute Febrile Illness, Sabah, East Malaysia

**DOI:** 10.3201/eid2607.191722

**Published:** 2020-07

**Authors:** Matthew J. Grigg, Timothy William, Emily G. Clemens, Kaajal Patel, Arjun Chandna, Christopher S. Wilkes, Bridget E. Barber, Nicholas M. Anstey, J. Stephen Dumler, Tsin W. Yeo, Megan E. Reller

**Affiliations:** Infectious Diseases Society Sabah–Menzies School of Health Research, Kota Kinabalu, Malaysia (M.J. Grigg, T. William, K. Patel, A. Chanda, C.S. Wilkes, B.E. Barber, N.M. Anstey, T.W. Yeo);; Menzies School of Health Research–Charles Darwin University, Darwin, Northern Territory, Australia (M.J. Grigg, K. Patel, A. Chanda, C.S. Wilkes, B.E. Barber, N.M. Anstey, T.W. Yeo);; Gleneagles Hospital, Kota Kinabalu (T. William);; Clinical Research Centre, Queen Elizabeth Hospital, Kota Kinabalu (T. William);; QIMR Berghofer Medical Research Institute, Brisbane, Queensland, Australia (B.E. Barber);; Uniformed Services University of the Health Sciences, Bethesda, Maryland, USA (J.S. Dumler);; Lee Kong Chian School of Medicine, Nanyang Technological University, Singapore (T.W. Yeo);; Communicable Disease Centre, Institute of Infectious Diseases and Epidemiology, Tan Tock Seng Hospital, Singapore (T.W. Yeo);; Duke University, Durham, North Carolina, USA (M.E. Reller);; Duke Global Health Institute, Durham (M.E. Reller)

**Keywords:** rickettsioses, *Orientia tsutsugamushi*, spotted-fever group rickettsiosis, typhus-group rickettsioses, acute febrile illness, tickborne diseases, Sabah, East Malaysia, vector-borne infections

## Abstract

*Orientia tsutsugamushi*, spotted fever group rickettsioses, and typhus group rickettsioses (TGR) are reemerging causes of acute febrile illness (AFI) in Southeast Asia. To further delineate extent, we enrolled patients >4 weeks of age with nonmalarial AFI in Sabah, Malaysia, during 2013–2015. We confirmed rickettsioses (past or acute, IgG titer >160) in 126/354 (36%) patients. We confirmed acute rickettsioses (paired 4-fold IgG titer rise to >160) in 38/145 (26%) patients: 23 *O. tsutsugamushi*, 9 spotted fever group, 4 TGR, 1 *O. tsutsugamushi*/spotted fever group, and 1 *O. tsutsugamushi*/TGR. PCR results were positive in 11/319 (3%) patients. Confirmed rickettsioses were more common in male adults; agricultural/plantation work and recent forest exposure were risk factors. Dizziness and acute hearing loss but not eschars were reported more often with acute rickettsioses. Only 2 patients were treated with doxycycline. Acute rickettsioses are common (>26%), underrecognized, and untreated etiologies of AFI in East Malaysia; empirical doxycycline treatment should be considered.

## Abstract 2

Because acute rickettsioses are common, underrecognized, and untreated etiologies of these illnesses, empirical doxycycline treatment should be considered.

Rickettsioses and related infections, including scrub typhus, caused by *Orientia tsutsugamushi*; spotted fever group rickettsioses (SFGR), often called tick typhus; and typhus group rickettsioses (TGR), also called murine typhus, are reemerging and neglected causes of acute febrile illness (AFI) in Southeast Asia (*1*–*4*). Serologic confirmation of acute rickettsioses, however, requires demonstration of a 4-fold rise in IgG titer by indirect fluorescent antibody (IFA) because acute-phase IgG might represent prior infection and IgM might indicate recent or cross-reactive antibody (*1*). Furthermore, serologic testing in the early phase of illness is not helpful because antirickettsial antibodies are often not present within the first 2 weeks.

Rickettsioses have been reported in Malaysia historically (*2*–*4*). Malaysia state tertiary hospital data for the period 1994–1999 identified antirickettsial IgG or IgM in 6,442 (10.6%) tested samples (IgG or IgM titer >400 by the indirect immunoperoxidase test), including 4.9% *O. tsutsugamushi*, 3.1% TGR, and 2.6% SFGR (*5*). Although rickettsioses are notifiable diseases according to Malaysia Ministry of Health guidelines (*6*), routine testing is not conducted, paired acute-phase and convalescent-phase serum samples are rarely available, and only 53 infections were reported nationally during 2009–2015 (*7*). In the East Malaysia state of Sabah, 2 cases were reported over that period despite a seroprevalence of 5.5% among 11,037 patients tested in previous years at the state tertiary referral hospital (*5*) and high (>50%) seropositivity reported in febrile patients from certain rural areas (*8*). We prospectively enrolled patients and performed reference standard diagnostic testing to document and describe the epidemiology and clinical features of confirmed rickettsioses among patients with nonmalarial AFI in Sabah, East Malaysia.

## Methods

### Study Sites and Referral System

We conducted the study during December 12, 2013–July 15, 2015, in 2 adjacent district referral hospitals, Kudat and Kota Marudu, in northwest Sabah, Malaysia, on the island of Borneo. These districts cover an area of 3,204 km^2^ and had an estimated population in 2015 of ≈169,000 (*9*). According to Malaysia Ministry of Health divisional health facility structures, each district has a single central referral hospital and subdistrict health clinics. The climate is tropical, has no defined dry season, and experiences increased rainfall during November–March. The region consists of both coastal and inland areas with elevations up to 1,000 m and extensive recent human land-use change from intact forest to oil palm plots and plantations (*10*).

### Study Population

Patients >4 weeks of age with a documented temperature of >38°C within 48 hours of admission to the medical or pediatric wards and a negative malarial blood film were eligible. We obtained consent from the patient or a parent or guardian if the patient was <18 years of age; we also obtained assent from persons 7–17 years of age.

### Study Procedures

Trained research nurses used standardized case record forms to collect structured epidemiologic, clinical, and laboratory data, including the admitting clinician’s findings on examination, provisional diagnosis, and management. At enrollment, venous blood was obtained for routine and research-related testing after consent. Patients were requested to return for routine hospital outpatient follow-up and convalescent-phase serum sampling 14 days after enrollment.

### Laboratory Procedures

Hematologic and biochemical tests, blood cultures, and malaria smears were performed in the hospital laboratory. Anemia was classified according to World Health Organization age- and sex-based criteria (*11*). Acute kidney injury was defined per KDIGO criteria (*12*).

We conducted IFA testing at the Uniformed Services University for the Health Sciences (Bethesda, MD, USA) to confirm rickettsial infections; 2 experts read each slide, as described (*13*,*14*). We screened convalescent-phase serum samples using IFA at 1:80 dilution for IgG to *O. tsutsugamushi* (Karp strain), SFGR (*Rickettsia conorii* Malish 7 strain), and TGR (*R. typhi*); for reactive samples, we tested acute- and convalescent-phase paired serum samples together and titrated positives to 2,560. To evaluate the utility of acute-phase IgM for identifying paired IgG-confirmed acute infections, we tested a subset with paired IgG results for IgM in addition to those without convalescent-phase serum. We screened acute-phase serum samples for IgM at 1:40 and titrated positives to 1,280.

We performed multiplex real-time PCR to detect *O. tsutsugamushi* (56-kDA major outer membrane protein gene), SFGR (consensus *ompA* sequence), and TGR (*R. typhi* 17-kDa lipoprotein precursor gene) on EDTA-anticoagulated blood or buffy coat stored at −70°C as described (*15*). We tested 5 µL of DNA extracted from a 200 µL sample of EDTA-anticoagulated whole blood or buffy coat; we required 2 out of 2 or 3 replicates to confirm a positive result by PCR.

### Case Definitions

We used stringent criteria to define rickettsial infections (*16*), as reported previously (*13*,*14*). We defined confirmed rickettsioses as any IgG titer >160; confirmed past rickettsial infection as stable or declining IgG titer >160; confirmed acute rickettsial infection as a >4-fold rise in IgG titer by paired IFA to convalescent-phase titer of >160, positive PCR result, or both; confirmed probable acute rickettsial infection as a 2-fold rise in IgG titer with convalescent-phase titer >160 or single IgG titer >160 without paired serum samples; and possible rickettsioses as any IgG titer of 80 (acute infection if IgG seroconversion). We distinguished SFGR from TGR when we observed a >2-fold difference in SFGR versus TGR titer; if titers were equal, we categorized the infection as group indeterminate.

### Statistical Analysis

We analyzed data by using Stata (StataCorp, https://www.stata.com). We performed 2-group comparisons by using the Student *t*-test for continuous variables with normal distributions or the Wilcoxon–Mann–Whitney test for skewed distributions. For categoric variables, we used a χ^2^ or Fisher exact test. We calculated odds ratios (ORs) and 95% CIs by using the Mantel-Haenszel method.

### Ethics

We obtained ethics approval for this study. The study was approved by relevant institutional review boards in Malaysia (National Medical Research Ethics Committee), Australia (Menzies School of Health Research), and the United States (John Hopkins University and Duke University).

## Results

**Figure 1:**
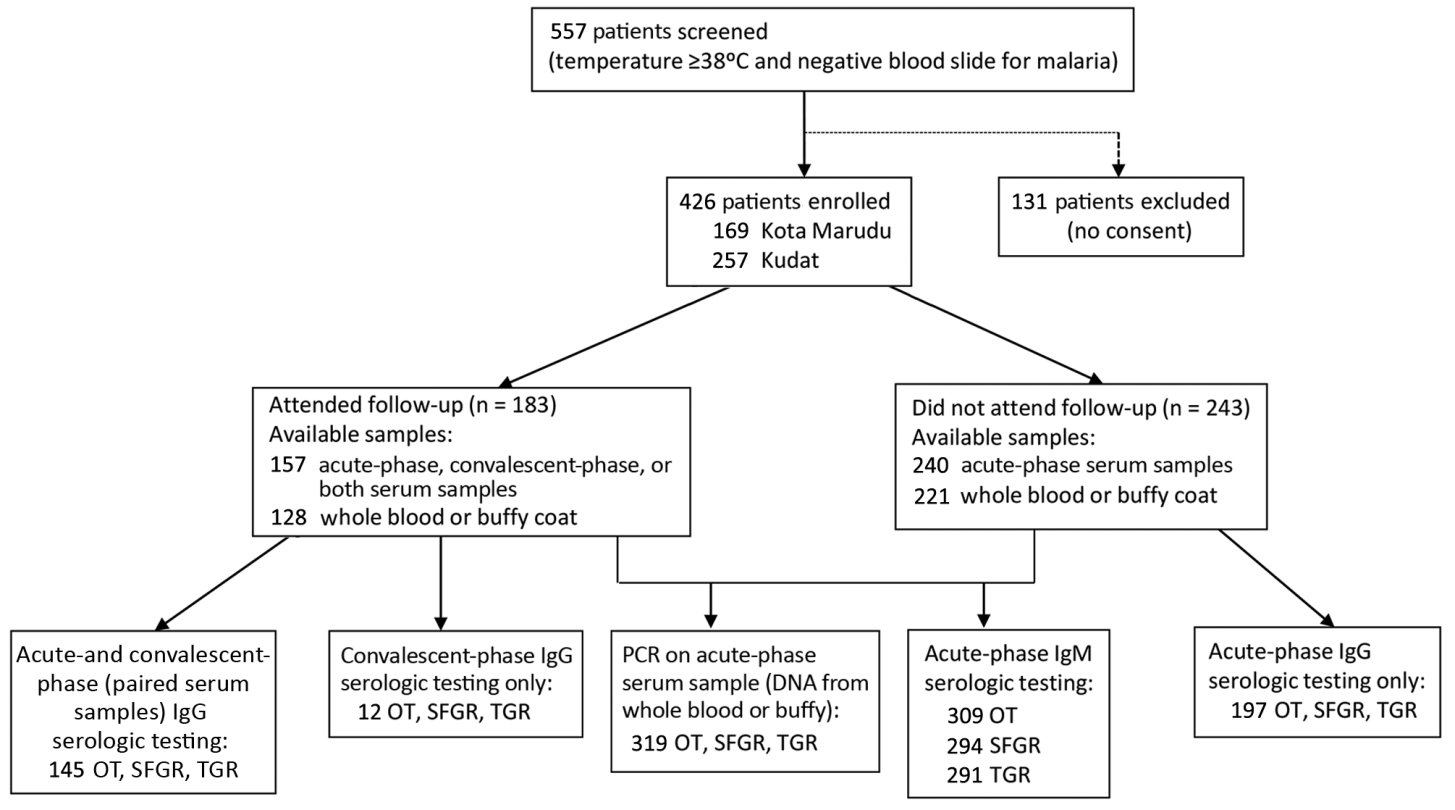
Enrollment flowchart and laboratory testing in a prospective cohort study of acute febrile illness attributable to rickettsioses, Sabah, East Malaysia, 2013–2015. OT, *Orientia tsutsugamushi*; SFGR, spotted-fever group rickettsiosis; TGR, typhus-group rickettsioses.

Of 557 patients who met eligibility criteria, 426 (77%) consented to enrolment and blood collection; 183 (43%) patients returned for convalescent follow-up (median 13 days; interquartile range (IQR) 12–14 days), and 157 (37%) had acute-phase serum samples, convalescent-phase serum samples, or both collected (Figure 1). Among the 243 patients who did not attend follow-up appointments, 240 had acute-phase serum samples available. A total of 354 patients had acute-phase serum samples, convalescent-phase serum samples, or both available to test for rickettsioses; of these, 145 (41%) patients had paired serum samples. Acute-phase whole blood or buffy coat was available for 319 patients. We observed no difference in median age or sex between those patients with and without paired serum samples.

### Serologic Diagnosis

Testing for IgG by IFA confirmed rickettsial infections in 126 of 354 (36%) patients, including 96 (27%) with scrub typhus (*O. tsutsugamushi* infection), 26 (7%) with SFGR, and 25 (7%) with TGR (Table 1); 6 patients had both *O. tsutsugamushi* and SFGR, 8 patients both *O. tsutsugamushi* and TGR, 1 patient both SFGR and TGR, and 3 patients *O. tsutsugamushi*, SFGR, and TGR IgG. We found confirmed past infection (stable or declining IgG titers >160) in 17 (12%) of 145 patients. Patients with serologic evidence of acute or past SFGR infections were older (median 60 years [IQR 45–73 years]) than patients with *O. tsutsugamushi* infection (median 38 years [IQR 24–57 years]; p = 0.003) but not patients with TGR (median 48 years [IQR 38–58 years]; p = 0.25) infections.

**Table 1:** Serologic and molecular detection of confirmed rickettsioses in a prospective cohort study of acute febrile illness attributable to rickettsioses, Sabah, East Malaysia, 2013–2015*

Confirmed rickettsial infections	OT	SFGR	TGR	Total
Confirmed age indeterminate (acute or past); acute-phase or convalescent-phase IgG titer >160,† n = 354	96 (27 [23–32])	26 (7 [5–11])	25 (7 [5–10])	126 (36 [31–41])
Confirmed past infection; acute-phase IgG titer >160 with stable or decreasing paired titer,‡ n = 145	13 (9 [5–14])	4 (3 [1–7])	4 (3 [1–7])	17 (12 [7–18])
Confirmed rickettsial infection, acute				
All acute confirmed,§ n = 378	26 (7 [5–10])	18 (5 [3–7])	7 (2 [1–4])	49 (13 [10–17])
>4-fold rise in IgG titer,§ n = 145	25 (17 [12–24])	10 (7 [4–12])	5 (3 [1–8])	38 (26 [20–34])
With seroconversion¶	13 (9 [5–14])	9 (6 [3–11])	2 (1 [<1–5])	22 (14 [9–20])
PCR positive,# n = 319	1 (<1 [0–2])	8 (2 [1–5])	2 (1 [0–2])	11 (3 [2–6])
Copy number/mL, mean	5,164	482	3477	
Confirmed rickettsial infection, probable acute				
All probable acute, n = 354	58 (16 [13–21])	12 (3 [2–6])	16 (5 [3–7])	77 (22 [18–26])
Paired serum samples				
2-fold IgG titer rise to >160,** n = 145	6 (4 [2–9])	1 (1 [<1–4])	2 (1 [<1–5])	7 (5 [2–10])
Single serum samples				
Acute-phase IgG >160,†† n = 197	45 (23 [18–29])	8 (4 [2–8])	12 (6 [4–10])	60 (30 [24–37])
Convalescent-phase IgG >160,‡‡ n = 12	7 (58 [32–81])	3 (25 [9–53])	2 (17 [5–45])	10 (83 [55–95])

*Values are no. (% [95% CI]) unless otherwise indicated. Total does not equal sum of individual infections because some of the individual infections are co-infections. OT, *Orientia tsutsugamushi*; SFGR, spotted-fever group rickettsiosis; TGR, typhus-group rickettsioses.  †Includes 18 patients with serologic evidence of OT/SFGR (6); OT/TGR (8); SFGR/TGR (1); OT/SFGR/TGR (3). ‡Includes 3 patients with serologic evidence of OT/TGR (2) and OT/SFGR/TGR (1). §Includes 2 co-infections: 1 patient positive for OT and SFGR (both with acute-phase IgG titer of 40 and convalescent-phase titer of 320) and 1 patient for OT and TGR (both with acute-phase IgG titer of 40 and convalescent-phase titer 160). ¶Includes 1 patient each with serologic evidence of OT/TGR and OT/SFGR. #No PCR-positive patients were confirmed also by paired IgG serology; 8 patients had acute-phase serum samples, and 1 TGR PCR-positive patient had IgM 2,560. **Includes 2 patients with 2-fold IgG titer rises to OT and TGR ††Includes 3 patients positive for OT and TGR and 2 patients positive for OT and SFGR ‡‡Includes 1 patient each with serologic evidence of SF/TG and OT/TG.

Among patients with paired serum samples, we confirmed 38 (26% [95% CI 20–34]) as having acute rickettsial infections based on a 4-fold rise in IgG titer, including 23 (15%) with *O. tsutsugamushi*, 9 (6%) SFGR, 4 (3%) TGR, and 2 co-infections (1 *O. tsutsugamushi*/SFGR and 1 *O. tsutsugamushi*/TGR [1% combined]). Most (22/38 [58%]) cases were associated with IgG seroconversion, including 13/25 (52%) acute *O. tsutsugamushi* infections, 9/10 (90%) acute SFGR, and 2/5 (40%) acute TGR.

Probable acute rickettsial infections were detected in 77/354 (22%) persons, including 58 with *O. tsutsugamushi*, 12 with SFGR, and 16 with TGR; 70 of these lacked paired serum samples. Among those with paired serum samples, sensitivity of acute-phase IgM for acute rickettsioses was 8% (95% CI 2%–21%) and specificity was 94% (95% CI 87%–98%). We observed possible acute rickettsioses in an additional 37/354 patients (10% [95% CI 7%–14%]) (Appendix Tables 1–3).

### Diagnosis by PCR

An additional 11 of 319 patients with acute-phase whole blood or buffy coat available had acute rickettsial infections by PCR. These infections were 1 *O. tsutsugamushi*, 8 SFGR, and 2 TGR (Table 1).

### Clinical and Laboratory Features of Acute Rickettsial Infections

Patients with confirmed acute rickettsial infections (n = 49; median age 39 years) were equally likely to be male and were of similar age (p = 0.32) to those with probable acute rickettsioses (median age 43 years); both groups were slightly older than those with no rickettsial infection (median age 29 years; p = 0.007) (Table 2). The median age of those with acute infections did not differ significantly (p = 0.69): 36 years (IQR 21–51 years) for *O. tsutsugamushi*, 49 years (IQR 12–58 years) for SFGR, and 38 years (IQR 27–56 years) for TGR. Eight children (<15 years of age) had confirmed acute rickettsial infections (5 SFGR, 2 *O. tsutsugamushi*, and 1 TGR), of whom 5 (63%) had their infections confirmed by PCR; the youngest was a 6-month-old infant. The median duration of hospitalization for those with confirmed acute rickettsial infections was 5 days (IQR 3–7 days), which was comparable to those without rickettsioses. No deaths were reported.

**Table 2:** Clinical features of patients with acute rickettsioses versus no rickettsioses in a prospective cohort study of acute febrile illness attributable to rickettsioses, Sabah, East Malaysia, 2013–2015*

Characteristic	Confirmed acute rickettsial infection	Probable acute rickettsial infection	p value for confirmed vs. probable acute infection	No rickettsial infection	p value for confirmed or probable acute vs. no infection	p value for confirmed acute vs. no infection
No. patients	49	77	NA	102	NA	NA
Demographics						
Age, median, y (IQR)	39 (2–56)	43 (28–62)	0.32	29 (8–55)	**0.007**	0.15
Child <15 y of age	8 (16)	3 (4)	**0.023**	29 (28)	**<0.001**	0.22
Sex						
M	31 (63)	48 (62)	0.92	48 (47)	**0.029**	0.20
F	18 (37)	29 (38)		54 (53)		
Symptoms						
Symptoms data available	48 (98)	75 (97)	0.99	102 (100)	0.99	0.99
Fever duration, median days (IQR)	2 (2–4)	3 (2–5)	0.46	3 (1–4)	0.64	0.89
Headache	36 (75)	60 (80)	0.65	60 (59)	**0.004**	0.05
Dizziness	33 (69)	44 (59)	0.23	49 (48)	**0.029**	**0.017**
Confusion	5 (10)	5 (7)	0.34	9 (9)	0.75	0.75
Vision changes	8 (17)	10 (13)	0.56	11 (11)	0.42	0.31
Retro-orbital pain	11 (23)	19 (25)	0.99	22 (22)	0.82	0.85
Hearing loss	7 (15)	4 (5)	0.11	2 (2)	**0.023**	**0.005**
Coryza	16 (33)	22 (30)	0.50	32 (31)	0.82	0.81
Cough	23 (48)	34 (45)	0.59	41 (40)	0.48	0.37
Dyspnea	16 (33)	13 (17)	**0.047**	27 (26)	0.64	0.39
Joint pain	20 (42)	29 (39)	0.62	36 (35)	0.57	0.45
Muscle pain	14 (29)	13 (17)	0.08	23 (23)	0.81	0.38
Lethargy	32 (67)	45 (60)	0.37	59 (58)	0.54	0.30
Nausea	16 (33)	34 (46)	0.16	44 (44)	0.71	0.23
Vomiting	13 (27)	27 (36)	0.32	47 (46)	**0.035**	**0.027**
Abdominal pain	20 (42)	33 (44)	0.56	41 (40)	0.48	0.86
Loss of appetite	34 (71)	49 (66)	0.43	70 (68)	0.76	0.79
Diarrhea	12 (25)	20 (27)	0.67	32 (31)	0.49	0.42
Dysuria	4 (8)	6 (8)	0.99	6 (6)	0.61	0.46
Signs						
Conjunctival suffusion	0/46 (0)	0/73 (0)	NA	2/95 (2)	0.21	0.99
Respiratory distress†	10 (21)	21 (27)	0.67	34 (33)	0.21	0.17
Respiratory crepitations on auscultation	5 (10)	12/74 (16)	0.25	18 (18)	0.32	0.33
Abnormal chest radiograph result	8/14 (57)	18/31 (58)	0.50	23/39 (59)	0.70	0.99
Maculopapular rash	4 (8)	10/75 (13)	0.37	6 (6)	0.23	0.73
Eschar	0/47 (0)	0/74 (0)	NA	0/101 (0)	NA	NA
Lymphadenopathy	0/46 (0)	2/72 (3)	0.53	3/98 (3)	0.67	0.55
Abdominal tenderness	7/44 (16)	5/49 (10)	0.36	11/91 (12)	0.76	0.59
Hepatomegaly	10 (21)	9/75 (12)	0.13	10 (10)	0.17	0.06
Splenomegaly	3/41 (7)	2/73 (3)	0.33	2/89 (2)	0.46	0.33

*Values are no. (%) unless indicated. Bold indicates a statistically significant difference (p<0.05). Results are from time of study enrolment unless indicated. IQR, interquartile range; NA, not applicable. †Respiratory rate >30 breaths/min or tissue oxygen saturation <96% on room air.

Clinical and laboratory features of probable acute rickettsial infections were comparable to those with confirmed infections (Table 2), except for less frequent dyspnea (17% vs. 33%; p = 0.047). Headache was more common in those with confirmed or probable acute rickettsial infection than those with no acute rickettsial infection (77% vs. 59%; p = 0.004), especially among those with SFGR or TGR (81%) compared with *O. tsutsugamushi* (76%) or neither (59%) (Appendix Tables 1–3). Dizziness was more frequent in those with confirmed and probable acute rickettsial infections (63%) than those with no rickettsial infection (48%; p = 0.029); however, vomiting was less common (32% vs. 46%; p = 0.035). Hearing loss was more common in those with acute (9%) compared with no rickettsial infection (2%; p = 0.023); this finding was most pronounced in confirmed *O. tsutsugamushi* infection (19% [95% CI 7%–39%]; p = 0.004) but also present in SFGR (18% [95% CI 4%–43%]; p = 0.021). Thirteen (11%) patients with acute rickettsioses had a maculopapular rash, but none had an eschar.

Provisional clinical diagnoses among patients with confirmed acute rickettsioses included 7 (14%) dengue, 6 (12%) acute undifferentiated fever, 6 (12%) community-acquired pneumonia, 5 (10%) urinary tract infection, 4 (8%) leptospirosis, 4 (8%) gastroenteritis, and 23 (47%) other diagnoses. No patient with or without confirmed rickettsial infection had a provisional diagnosis of acute rickettsial infection.

Anemia occurred in 24 (51%) patients with acute rickettsioses and hematologic results available, and we noted a hemoglobin level of <80 g/L in 3 adults (Appendix Table 4). Peripheral leukocyte counts, renal function after controlling for age, and acute kidney injury, observed in 6/33 patients (18%), were similar in patients with and without acute rickettsioses.

Doxycycline was administered to 2 (4%) patients with confirmed acute rickettsioses (both *O. tsutsugamushi* infections), albeit in both cases for a provisional diagnosis of gastroenteritis. None of the 6 rickettsial case-patients with a provisional diagnosis of acute undifferentiated fever were treated with doxycycline.

### Epidemiologic Features of Patients with Confirmed Acute or Past Rickettsial Infections

Patients with acute or past rickettsial infections were older than those without (median age 43 vs. 24 years; p<0.001); however, the distribution by sex was similar (57% male among those with past rickettsial infection vs. 52% among those without rickettsial infection). Most patients (67%) reported rural residence (Appendix Table 5). Recently spending time in forest areas was more common (OR 2.1 [IQR 1.0–4.3]; p = 0.037) in patients with acute or past rickettsial infection (14%) than patients with neither (7%), including staying overnight in the forest for those with scrub typhus (11%; p = 0.047). Patients were more likely to report a primary occupation as a rubber tapper (OR 5.9 [IQR 1.9–18.5]; p = 0.002) for those with acute or past rickettsial infections (10%) than for patients without rickettsioses (2%). Of the 13 rubber tappers with seroprevalent rickettsioses, 3 (23%) had confirmed acute scrub typhus and 5 (38%) past scrub typhus. Farmers were also more likely (OR 2.8 [IQR 1.0–7.3]; p = 0.041) to have (8%) than not to have (3%) seroprevalent rickettsial infections. Patients who were unemployed had an increased risk of rickettsioses (OR 1.9 [IQR 1.0-3.5]; p = 0.042), of whom 4/24 had a recent travel history. No other occupations were associated with an increased risk of seroprevalent rickettsioses.

## Discussion

**Figure 2:**
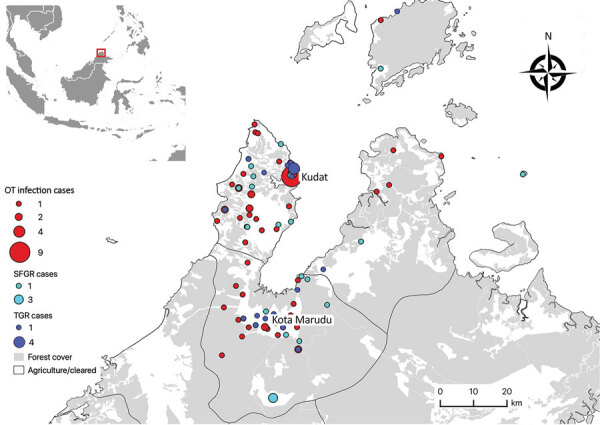
Village-level geographic distribution of confirmed acute and probable acute rickettsioses cases in a prospective cohort study of acute febrile illness attributable to rickettsioses, Sabah, East Malaysia, 2013–2015. Inset map shows study area in Sabah, Malaysia. OT, *Orientia tsutsugamushi* infection; SFGR, spotted-fever group rickettsiosis; TGR, typhus-group rickettsioses.

Although rickettsial infections were first documented in Malaysia in 1925 (*17*), few cases are currently confirmed and reported nationally (*18*). Paired serum samples are infrequently obtained, and IFA requires expertise; both these factors limit diagnostic efforts. By confirming acute infection based on a 4-fold rise in IgG titer, PCR positivity, or both, we documented and characterized rickettsioses as an important cause of nonmalarial AFI in Malaysia (Figure 2). We found definitive serologic evidence of acute or past rickettsioses in 36% of patients and confirmed acute infections in 26% of patients. Scrub typhus (*O. tsutsugamushi* infection) was the most common rickettsiosis, especially among rural residents. 

Acute-phase IgM demonstrated insufficient sensitivity for identifying acute rickettsioses. Moreover, acute-phase IgG, which cannot distinguish acute from past rickettsioses, is an imperfect measure of seroprevalence because antibodies decline over time even in patients who are not treated (*19*). PCR also detected few acute infections compared with paired serologic tests. Rickettsial infections were unsuspected clinically, and nearly all were untreated. Therefore, increased clinical awareness, improved diagnostic tools, and empirical use of doxycycline are essential to reduce illness and death attributable to rickettsioses.

Confirmation of acute rickettsioses as a major cause of AFI in Sabah, Malaysia, is to be expected because rickettsioses have been identified elsewhere in the region. A large multicenter study in Thailand, Vietnam, and Indonesia showed that 6% of 1,578 enrolled patients had serologically confirmed acute rickettsioses (*20*). Other rigorous clinical and cross-sectional studies have demonstrated marked spatial heterogeneity in seroprevalence of rickettsioses (*21*), including 1% in Thailand (*22*), 33% in areas bordering Myanmar (*23*), 7% to 27% in Laos (*24*–*26*), and up to 10% in parts of Indonesia (*27*,*28*). Our study suggests that rickettsioses are at least as common (26%) in Malaysia as elsewhere in the region.

Rickettsioses are likely to be reemerging in East Malaysia. Despite the presence of a common vector of scrub typhus, *Leptotrombidium (L.) deliense* mites, being documented in coastal areas for decades (*29*), a single statewide study (including Kudat District) in the 1980s reported no acute rickettsioses among 383 febrile patients with paired serum samples tested by IFA (*30*). Sero-epidemiologic findings from 837 persons at that time demonstrated a low seroprevalence of 0.8% for *O. tsutsugamushi*, 8.6% for SFGR, and 0% for TGR (*30*). At the Sabah state tertiary referral hospital during 1994–1999, a total of 11,037 samples from febrile patients sent for acute IgG or IgM testing showed a comparatively low seroprevalence of 2.6% for *O. tsutsugamushi*, 1.3% for TGR, and 1.6% for SFGR (*5*). However, the inability to confirm acute rickettsioses, even retrospectively, if paired serum samples are not obtained has constrained broader public health understanding of their epidemiology and disease prevalence among the at-risk population in Sabah.

The epidemiology of rickettsial infections in this study is consistent with previous reports of scrub typhus and spotted fever rickettsioses described in rural areas in Southeast Asia (*31*); those reports indicated that infections were more commonly found in male adults, in agricultural, plantation, or forestry workers (*8*,*28*,*32*,*33*), and in persons with lower socio-economic status (*21*,*34*). The presence of typhus group rickettsioses in rural residents in our study population, despite generally being reported more frequently in urban populations (*31*), highlights the need for further studies to better understand the epidemiology of rickettsioses in Malaysia and to design appropriate control measures. Because SFGR and TGR cross-reactivity by IFA is expected, testing for all 3 rickettsioses (*O. tsutsugamushi* infection, SFGR, and TGR) is important.

Factors explaining a potential increase in rickettsioses in Sabah could in part be related to changes in human land use (*10*) and the relationship with occupational or travel behavior and vector bionomics (*35*). The effect of habitat on *O. tsutsugamushi* seroprevalence has previously been evaluated in peninsular Malaysia; higher seropositivity overall was observed in indigenous adults with exposure to intact forest areas (73%) compared with forest fringe (48%) and village areas (8%) (*4*). These findings are broadly consistent with the association in our study between persons with acute or past rickettsioses and a history of extended forest exposure. Rubber plantation workers in peninsular Malaysia have been reported to have higher SFGR prevalence (40%), compared with *O. tsutsugamushi* (8%) and TGR (1%) (*36*). In contrast, although those identified as rubber tappers in our study had high seroprevalence to rickettsioses overall, most of these infections were attributable to *O. tsutsugamushi*.

We found that patients with acute rickettsioses had clinical findings similar to those without acute rickettsioses, with the exception of more frequent dizziness and hearing loss and less frequent vomiting. Detection of hearing loss might assist in clinical recognition of rickettsioses and has been reported in up to one third of patients with acute scrub typhus (*37*) as well as in those with SFGR (*38*). Eschar occurs most frequently on the anterior chest in women and the groin in men with scrub typhus (*39*); the relative rarity of eschar and higher frequency of hepatomegaly found in our study was also previously described in Malaysia (*2*). We observed severe cases of acute kidney injury in a small number of patients; however, acute kidney injury remains clinically important because of the subsequent increased risk for chronic kidney disease (*40*).

Common laboratory markers, such as abnormalities in leukocyte count, thrombocytopenia, anemia, and raised liver transaminases, were all nonspecific (*31*). Neutropenia occurred in 1 patient with acute scrub typhus, despite this sign being reported as a potentially useful clinical tool to differentiate scrub typhus from dengue (*41*). AFI was primarily attributed to dengue by treating clinicians, who initiated dengue fluid management protocols. In other cases, empiric treatment of suspected typhoid or leptospirosis was initiated with cephalosporins or penicillins, which are ineffective against rickettsioses. Treatment with doxycycline is recommended by the Malaysia Ministry of Health (*42*) because an estimated 6% of untreated scrub typhus cases in Asia are fatal (*43*); however, we found that rickettsioses were unsuspected and untreated.

Tools to support early diagnosis and local evidence-based clinical algorithms to guide directed and empirical treatment of undifferentiated AFI are needed. Diagnosis of acute rickettsioses remains problematic. *Orientia* and *Rickettsia* spp. are obligate intracellular bacteria predominantly targeting endothelial cells (*44*,*45*); hence, real-time PCR on peripheral blood is insensitive for the detection of acute rickettsioses (*15*,*19*,*46*). Incomplete follow-up meant the sensitivity of PCR could not be directly measured against gold-standard IFA serologic diagnosis for SFGR and TGR, although low PCR sensitivity of 18% has been reported (*19*). Longstanding technical difficulties with IFA also include the need for extensively trained readers and expensive equipment. Conventional use of antibody detection against only 3 major *O. tsutsugamushi* antigen groups and serologic cross-reactivity further limits accurate diagnoses; however, high antigenic variation has been observed across Southeast Asia (*47*,*48*). We found that acute-phase IgM was a poor predictor of acute rickettsial infection even in the setting of acute scrub typhus, underscoring the importance of increased clinical awareness, improved diagnostic tools, and empiric treatment of those with epidemiologic or clinical risk factors.

The main limitation of this study was the relatively low proportion (41%) of patients with both acute-phase and convalescent-phase serum samples compared with our previous studies (*17*,*18*). For this reason, we also performed PCR and tested for IgM, even though sensitivity of the former and specificity of the latter are limited for identifying acute rickettsioses (*19*). Our estimates of the seroprevalence of rickettsioses and incidence of confirmed acute rickettsioses are conservative. First, only half of patients returned for convalescent follow-up, yet most confirmed acute rickettsioses were seroconversions rather than based on 4-fold rises in titer alone. Second, over half of patients who did have a convalescent-phase serum sample obtained had it obtained earlier than the recommended >14 days. In experimental human SFGR infection, >3 weeks were required before the geometric mean titer of infected volunteers reached diagnostic IgG titers (*49*). Third, antibody detection of different antigen groups such as that observed in *O. tsutsugamushi* was based on cross-reactivity to the single Karp strain. Finally, patients with seroconversions indicative of past infection might be missed by a screening strategy that entails testing convalescent-phase serum samples first.

To better estimate the actual prevalence of acute rickettsioses, we additionally delineated patients with confirmed rickettsioses with likely acute infection (patients with rising or single IgG titers of >160) as having probable acute infections. The likely underestimation of acute infections caused by diagnostic uncertainty is supported by the similarity in initial clinical and laboratory findings between confirmed and probable acute rickettsioses. The burden and clinical features of rickettsioses are likely underestimated in our study not only because of underreporting of laboratory-confirmed cases but also because persons with mild illness, nonspecific features, and limited physical or economic ability to travel to hospital and persons living in remote areas might not have sought care. Similarly, persons with severe disease might have sought care at or been transferred to a tertiary hospital before study enrollment or died before care they sought or obtained care. TGR prevalence is also potentially underestimated because our population was mainly rural rather than urban. However, our data are likely generalizable to similar populations in Sabah and potentially neighboring areas.

In conclusion, rickettsioses are prevalent among persons with nonmalarial AFI in Sabah, Malaysia. A comprehensive prospective study of rickettsial infections across Malaysia is warranted to define the totality of regional risk. Improved diagnostic tools are urgently needed; in their absence, increased clinical suspicion and empirical doxycycline might avert disease and death attributable to rickettsioses.

Appendix Additional information about rickettsioses as major etiologies of unrecognized acute febrile illness, Sabah, East Malaysia.
